# Integration of* Andrographis paniculata* as Potential Medicinal Plant in Chir Pine (*Pinus roxburghii* Sarg.) Plantation of North-Western Himalaya

**DOI:** 10.1155/2016/2049532

**Published:** 2016-08-01

**Authors:** Chandra Shekher Sanwal, Raj Kumar, S. D. Bhardwaj

**Affiliations:** ^1^Indian Forest Service, Uttarakhand Forest Department, Dehradun 248001, India; ^2^ICAR-IISWC, Research Center, Vasad, Anand, Gujarat 388306, India; ^3^Department of Silviculture and Agroforestry, Dr. Y. S. Parmar University of Horticulture and Forestry, Nauni, Solan, Himachal Pradesh 173 032, India

## Abstract

The integration of* Andrographis paniculata* under* Pinus roxburghii* (Chir pine) plantation has been studied to evaluate the growth and yield for its economic viability and conservation. It was grown on three topographical aspects, namely, northern, north-western, and western, at a spacing of 30 cm × 30 cm, followed by three tillage depths, namely, minimum (0 cm), medium (up to 10 cm), and deep (up to 15 cm) tillage. The growth parameters, namely, plant height and number of branches per plant, were recorded as significantly higher on western aspect and lowest on northern aspect except for leaf area index which was found nonsignificant. However under all tillage practices all the growth parameters in both understorey and open conditions were found to be nonsignificant except for plant height which was found to be significantly highest under deep tillage and lowest under minimum tillage. The study of net returns for* Andrographis paniculata* revealed that it had positive average annual returns even in understorey conditions which indicate its possible economic viability under integration of Chir pine plantations. Hence net returns can be enhanced by integrating* Andrographis paniculata* and this silvimedicinal system can be suggested which will help utilizing an unutilized part of land and increase total productivity from such lands besides conservation of the* A. paniculata in situ*.

## 1. Introduction

*Andrographis,* also known as “King of Bitters,” is a member of the plant family Acanthaceae. The leaves and stems of the plant are used to extract the active phytochemicals. The most medicinally active phytochemical is andrographolide.* Andrographis paniculata* has been reported as having antibacterial, antifungal, antiviral, choleretic, hypoglycemic, hypocholesterolemic, and adaptogenic effects [1]. Due to its “blood purifying” activity it is recommended for use in cases of leprosy, gonorrhea, scabies, boils, skin eruptions, and chronic and seasonal fevers [2]. A primary modern use of* A. paniculata* is for the prevention and treatment of the common cold. It appears to have antithrombotic actions, suggesting a possible benefit in cardiovascular disease [3]. Pharmacological and clinical studies suggest the potential for beneficial effects in diseases like cancer [4] and HIV infections [5]. Most of medicinal plants, even today, are collected from the wild. The continued commercial exploitation of these plants has resulted in receding the population of many species in their natural habitat. Consequently, cultivation of these plants is urgently needed to ensure their availability to the industry as well as to people associated with traditional system of medicine.

The Himalayan Subtropical Pine Forests are the largest in the Indo-Pacific region. They stretch throughout most of the 3,000-km length of the world's youngest and highest mountain range, the Himalayas. The dominant species in this belt of subtropical pine forest is Chir pine. Although Champion and Seth [6] indicate the presence of large areas of Chir pine in Arunachal Pradesh, the easternmost extent of large areas of Chir pine is in Bhutan. Thus Chir pine is a native of the interranges and principal valleys of the Himalaya; beginning from Afghanistan in the west and ending in Bhutan in the east it extends through Pakistan, India, and Nepal. In India its forests are found in Jammu and Kashmir, Haryana, Himachal Pradesh, Uttarakhand, parts of Sikkim, West Bengal, and Arunachal Pradesh. Compared with the adjacent broadleaf forests, this ecoregion is neither exceptionally rich in species nor high in endemic species. The total area under Chir forests is estimated to be 8,90,000 hectares and occurs between 450 m and 2300 m altitude.

Chir pine is the fastest growing among the conifers found in the Himalayas. The species is hardy, frugal in its soil requirements, and adapted to degraded sites which are deficient in nutrients. It grows with ease both on deep soils which should be well drained and on skeletal soils. Being a light demanding species, it easily rehabilitates exposed sites where most of broad leaved species rarely succeed. Chir pine being highly resistant to fire is better suited for tracts where complete fire protection is difficult to ensure. But the Chir pine forest has continued to be a neglected area for the cultivation of medicinal and aromatic plants. Also there is scanty information on the role and development of medicinal plant wealth as a part of Chir pine forest ecosystem. On the other hand with dwindling supplies from natural resources and increasing global demand, there is need to cultivate medicinal and aromatic plants (MAPs) to ensure their regular supplies as well as conservation. So for promoting their cultivation and conservation several approaches are feasible such as integrating MAPs as lower strata species in multistrata systems, cultivating short cycle medicinal and aromatic plants as intercrops in existing stands of plantation tree crops, and new forest plantations. Hence considering the medicinal importance of Kalmegh (*Andrographis paniculata*) for human health and well-being, present studies were undertaken on the possibility of raising Kalmegh (*Andrographis paniculata*) in Chir pine plantation of north-western Himalaya.

## 2. Material and Methods

The investigations were carried out at different aspects in Dr. Y. S. Parmar University of Horticulture and Forestry, Nauni, Solan, Himachal Pradesh. The experimental site is located within 30°51′N latitude and 76°11′E longitude (survey of India Toposheet number 55 F/1) at an elevation of 1250 m above mean sea level. The climate of the area is transitional between subtropical and subtemperate with maximum temperatures rising up to 37.8°C during summer. The mean annual temperature is 19.8°C. In general May and June are the hottest months whereas December and January are the coldest ones. The annual rain fall ranges between 800 and 1300 mm 75 percent is received during mid-June to mid-September. Growth and yield of* Andrographis paniculata* integrated under Chir pine and without Chir pine (open) on different aspects and under different tillage practices were studied separately. Hence, studies involved three factors, that is, aspects, tillage practices, and systems (crop grown in understorey of Chir pine and in open conditions).* A. paniculata* was grown on three aspects, namely, northern (A_1_), north-western (A_2_), and western (A_3_), at a spacing of 30 cm × 30 cm, followed by three tillage depths, namely, minimum (T_1_: 0 cm), medium (T_2_: up to 10 cm), and deep (T_3_: up to 15 cm) tillage. The 18 treatments, including all possible combination of three aspects, three tillage depths, and two systems, were used for the evaluation of* Andrographis paniculata* performance under three replicates in randomized block design.

Experimental field was prepared by removing the pine needles and tillage practices were done just before the onset of monsoon. Plots were prepared as per the treatment details under different tillage practices. The whole experiment was conducted under rainfed conditions entirely dependent on the monsoon rains. Keeping in view the forest site conditions no irrigation and fertilizer were applied and the selection of* Andrographis paniculata* as medicinal plant was done on the basis of its minimum input requirement for irrigation and fertilizers. For the transplanting of seedlings nursery was prepared and, with the commencement of monsoon and after getting the sufficient moisture availability in the soil in first fortnight of July, healthy seedlings of* A. paniculata* were lifted from nursery and transplanted in the experimental field. The crop was harvested in the month of December. The observations on growth parameters (height, number of branches per plant, and leaf area index) were recorded at vegetative, prebloom, and harvesting stage, whereas the data for the yield and the yield attributes were measured at the time of harvesting. To make the economic appraisal for integration of* Andrographis paniculata* with* Pinus roxburghii*, the yield of* Andrographis paniculata* was subjected to economic analysis by calculating cost of cultivation and gross and net returns per hectare.

### 2.1. Growth and Yield Attributes of Chir Pine

Average height, average diameter, and average value of crown area of* Pinus roxburghii *tree have been presented in Table 1. Average volume of the tree (Chir pine) was calculated using local volume table based on diameter (Solan forest division). Annual increment in volume thus calculated was then used to estimate returns from tree at the prevailing market price. The same procedure was used for calculating net returns from tree on all the three aspects. To make the economic appraisal for silvimedicinal systems the yield of* A. paniculata* was subjected to economic analysis by calculating cost of cultivation and gross and net returns per hectare. The entire data generated from the present investigation were analysed statistically using the technique of analysis of variance for factorial randomized design in accordance with the procedure outlined by K. A. Gomez and A. A. Gomez [7].

## 3. Results and Discussion

The year-wise data pertaining to the growth and yield attributes of* Andrographis paniculata*, as influenced by three topographical aspects and three tillage practices in both understorey of Chir pine and open conditions, have been explained and discussed here.

### 3.1. Growth of* A. paniculata*

The plant height, number of branches per plant, and leaf area index declined markedly in understorey of Chir pine as compared to open conditions. In all the growth stages plant height and number of branches per plant were recorded as significantly higher on western aspect and lowest on northern aspect except for leaf area index which was found nonsignificant (Table 2). However under all tillage practices all the growth parameters in both understorey and open conditions were found to be nonsignificant except for plant height which was found to be significantly highest under deep tillage and lowest under minimum tillage (Table 4). This infers that growth of* A. paniculata* reduced under Chir pine was due to low light intensity and increased competition for moisture and nutrients.

### 3.2. Yield of* A. paniculata*

The yield in* A. paniculata also* evinced a conspicuous decline in understorey of Chir pine compared to open conditions. However the effect of topographical aspect and tillage depth on yield was found nonsignificant in both understorey and open conditions (Tables 3 and 5). Similar trend was found with respect to below-ground dry weight. The yield reduction (13.01%) in understorey could be because of the lower value of plant height, number of branches, and below-ground dry weight on all aspect in understorey condition than the open condition. Thus the significantly lower value of all growth characters and yield in understorey conditions than open conditions can be attributed to adverse effect of tree canopy and intensity of shade in understorey condition compared to the open conditions. It is reported that reduction in yield and yield attributes of agricultural crop (rice) due to reduced intensity of light is not expressed solely through photosynthesis but may also be expressed through such indirect plant growth function as root growth [8]. Similar findings were reported by Pandey et al. [9] and Wardlaw [10] due to the decreased carbohydrate allocation to roots under shaded conditions. Thus the crop also suffered in terms of nutrient and water uptake, which though may be present in sufficient quantity, but could not be utilized by the intercrop due to reduced growth of root system.

Further the nonsignificant effect on yield might be attributed to nonsignificant differences in case of leaf area index and below-ground dry weight among different treatment combinations. The values for all growth parameters and yield attributes in all the seven medicinal and aromatic plants on all aspects were higher in open conditions than the understorey of Chir pine. This suggests that the plants grown in the open field as sole crop have better opportunities to reap more solar energy for photosynthetic activity and less intraspecific competition for critical resources like water, nutrients, and photosynthetically active radiations. These favorable factors seem to result in higher values of growth parameters in medicinal and aromatic crops in the open conditions. Chauhan [11], Karikalan et al. [12], and Thakur and Singh [13] have earlier made similar observations for different agricultural crop under agroforestry system. Apart from the above the lower values of grown parameters and yield attributes in understorey of* Pinus roxburghii* might be because of the possibility of accumulation of phytotoxin in soil over number of years, which might lead to allelopathic interaction with crops that cannot be ruled out.

### 3.3. Bioeconomic Appraisal of* A. paniculata*

The total operational cost has been found different among the treatments which includes total cost of agronomical practices and other variable and fixed cost components. In the silvimedicinal system the maximum net returns ($ 152.50 ha^−1^) were observed for crop growing on western aspect under deep tillage in understorey conditions followed by plots having crop ($ 135.07 ha^−1^) on western aspect under medium tillage in understorey conditions (Table 6). The minimum net returns ($. 40.57 ha^−1^) were obtained for crop growing on northern aspect under minimum tillage in open conditions. In all different treatment combinations higher net returns were observed in understorey of Chir pine compared to open conditions.

The study of net returns for* Andrographis paniculata* revealed that it had positive average annual returns. The positive net returns in case of* Andrographis paniculata* may be due to their suitability in the environment given in open conditions and in association with the Chir pine. This finding can be supported by Harrington et al. [14] who initiated a research to determine the separate effects of above- and below-ground competition and needlefall from overstorey pines on understorey plant performance and found that depending on species the effects of needlefall were positive, negative, or negligible. Further the positive net returns in* Andrographis paniculata* are attributed to lower cost of cultivation than gross returns. The lower cost of cultivation in the given condition can be due to three distinct reasons, namely, the lower rental value of land in association with Chir pine in understorey and open conditions, no use of fertilizers and irrigation practices, and lower cost of planting material, as the nursery was prepared for* Andrographis paniculata.*

## 4. Conclusion

Thus the successful integration of* Andrographis paniculata* can be recommended only in the wasteland having lower rental value like land in association with Chir pine (*Pinus roxburghii*). The* Andrographis paniculata* could better flourish on natural ecosystem under* in situ* conditions and the conservation and cultivation of these species under controlled cultural practices did not prove to be economically feasible under* ex situ* conditions. This suggests that Kalmegh can be integrated into the Chir pine forest and this will be equally true for the new afforestation areas.

## Figures and Tables

**Table 1:** Height, diameter, and crown area of *Pinus roxburghii* in the study area.

Aspect	Average height (m)	Average diameter (cm)	Crown area (m^2^)
A_1_: northern aspect	11.23	19.94	2.99
A_2_: north-western aspect	10.22	18.15	2.87
A_3_: western aspect	11.09	19.39	2.98

**Table 2:** Effect of aspect on plant height, number of branches per plant, and LAI of *Andrographis paniculata* in understorey and open conditions.

Aspect	Plant height (cm)									Number of branches per plant						Leaf area index (LAI)					
	Vegetative stage			Prebloom stage			Harvesting stage			Prebloom stage			Harvesting stage			Prebloom stage			Harvesting stage		
	Understorey	Open	Mean	Understorey	Open	Mean	Understorey	Open	Mean	Understorey	Open	Mean	Understorey	Open	Mean	Understorey	Open	Mean	Understorey	Open	Mean
A_1_: northern	21.70	24.34	23.02^a^	37.88	40.61	39.24^a^	43.33	46.29	44.81^c^	7.75	8.59	8.17^c^	13.06	14.03	13.54^b^	0.29	0.30	0.29^c^	0.45	0.46	0.45^c^
A_2_: north-western	22.55	25.42	23.98^a^	39.63	42.37	41.00^a^	44.30	48.56	46.43^b^	8.83	9.82	9.32^b^	14.13	15.57	14.85^ba^	0.30	0.31	0.30^b^	0.47	0.48	0.47^b^
A_3_: western	23.97	26.43	25.20^a^	41.31	44.12	42.71^a^	46.32	51.22	48.77^a^	9.90	10.89	10.39^a^	15.58	16.78	16.18^a^	0.31	0.32	0.31^a^	0.49	0.49	0.49^a^

Mean	22.74^b^	25.40^a^		39.61^b^	42.37^a^		44.65^b^	48.36^a^		8.83^b^	9.77^a^		14.26^b^	15.46^a^		0.30^b^	0.31^a^		0.47^b^	0.48^a^	

Different letters indicate significant differences at *p* < 0.05.

**Table 3:** Effect of aspect on biomass and yield (t/ha) of *Andrographis paniculata *in understorey and open conditions.

Aspect	Above ground fresh weight (t/ha)			Above ground dry weight (t/ha)			Below ground fresh weight (t/ha)			Below ground dry weight (t/ha)		
	Understorey	Open	Mean	Understorey	Open	Mean	Understorey	Open	Mean	Understorey	Open	Mean
A_1_: northern	1.00	1.26	1.13^a^	0.57	0.68	0.62^a^	0.15	0.19	0.17^a^	0.08	0.11	0.10^a^
A_2_: north-western	1.16	1.39	1.27^a^	0.63	0.73	0.68^a^	0.18	0.21	0.20^a^	0.10	0.12	0.11^a^
A_3_: western	1.51	1.72	1.62^a^	0.81	0.91	0.86^a^	0.23	0.26	0.25^a^	0.14	0.15	0.14^a^

Mean	1.23^b^	1.45^a^		0.67^b^	0.77^a^		0.19^b^	0.22^a^		0.11^b^	0.13^a^	

**Table 4:** Effect of tillage practices on height, number of branches per plant, and LAI of *Andrographis paniculata *in understorey and open conditions.

Tillage	Plant height (cm)									Number of branches per plant						Leaf area index (LAI)					
	Vegetative stage			Prebloom stage			Harvesting stage			Prebloom stage			Harvesting stage			Prebloom stage			Harvesting stage		
	Understorey	Open	Mean	Understorey	Open	Mean	Understorey	Open	Mean	Understorey	Open	Mean	Understorey	Open	Mean	Understorey	Open	Mean	Understorey	Open	Mean
T_1_: minimum	18.73	20.83	19.78^c^	35.75	38.86	37.30^a^	40.42	43.89	42.15^c^	7.05	7.26	7.15^c^	12.52	13.82	13.17^a^	0.28	0.29	0.28^a^	0.45	0.46	0.45^c^
T_2_: medium	24.30	27.08	25.69^b^	40.13	42.38	41.25^a^	44.49	47.84	46.16^b^	9.09	10.07	9.58^b^	14.65	15.72	15.18^a^	0.30	0.31	0.30^a^	0.47	0.48	0.47^b^
T_3_: deep	25.19	28.28	26.73^a^	42.94	45.86	44.40^a^	49.04	53.34	51.19^a^	10.13	11.26	10.69^a^	15.61	16.83	16.22^a^	0.31	0.32	0.31^a^	0.48	0.49	0.48^a^

Mean	22.74^b^	25.40^a^		39.61^b^	42.37^a^		44.65^b^	48.36^a^		8.83^b^	9.77^a^		14.26^b^	15.46^a^		0.30^b^	0.31^a^		0.47^b^	0.48^a^	

Different letters indicate significant differences at *p* < 0.05.

**Table 5:** Effect of tillage practices on biomass and yield (t/ha) of *Andrographis paniculata *in understorey and open conditions.

Aspect	Above ground fresh weight (t/ha)			Above ground dry weight (t/ha)			Below ground fresh weight (t/ha)			Below ground dry weight (t/ha)		
	Understorey	Open	Mean	Understorey	Open	Mean	Understorey	Open	Mean	Understorey	Open	Mean
T_1_: minimum	1.00	1.20	1.10^a^	0.54	0.64	0.59^a^	0.15	0.19	0.17^a^	0.09	0.10	0.10^a^
T_2_: medium	1.24	1.48	1.36^a^	0.68	0.78	0.73^a^	0.19	0.23	0.21^a^	0.11	0.13	0.12^a^
T_3_: deep	1.44	1.69	1.56^a^	0.79	0.90	0.84^a^	0.22	0.26	0.24^a^	0.13	0.15	0.14^a^

Mean	1.23^b^	1.45^a^		0.67^b^	0.77^a^		0.19^b^	0.22^a^		0.11^b^	0.13^a^	

Different letters indicate significant differences at *p* < 0.05.

**Table 6:** Bioeconomic appraisal of *Andrographis paniculata*.

Treatment combinations	*Andrographis paniculata*		
	Gross return ($)	Cost of cultivation ($)	Net return ($)
A_1_T_1_S_1_	186.28	118.92	67.37
A_1_T_2_S_1_	214.93	137.23	77.68
A_1_T_3_S_1_	253.18	147.20	105.98
A_1_T_1_S_0_	176.40	135.83	40.57
A_1_T_2_S_0_	200.25	151.88	48.37
A_1_T_3_S_0_	237.90	168.97	68.93
A_2_T_1_S_1_	193.55	127.72	65.83
A_2_T_2_S_1_	234.65	145.62	89.03
A_2_T_3_s_1_	258.35	161.70	96.65
A_2_T_1_S_0_	180.45	145.37	35.08
A_2_T_2_S_0_	223.50	161.77	61.73
A_2_T_3_S_0_	256.50	179.03	77.47
A_3_T_1_S_1_	242.05	144.83	97.22
A_3_T_2_S_1_	293.35	158.28	135.07
A_3_T_3_S_1_	329.65	177.15	152.50
A_3_T_1_S_0_	219.30	160.38	58.92
A_3_T_2_S_0_	285.00	179.60	105.40
A_3_T_3_S_0_	316.65	199.53	117.12

Note: one US dollar is equivalent to 60 Indian rupees.

A_1_: northern aspect; A_2_: north-western aspect; A_3_: western aspect.

T_1_: minimum tillage; T_2_: medium tillage; T_3_: deep tillage.

S_1_: understorey; S_0_: open.
